# Anti-cancer activity of the novel 2-hydroxydiarylamide derivatives IMD-0354 and KRT1853 through suppression of cancer cell invasion, proliferation, and survival mediated by TMPRSS4

**DOI:** 10.1038/s41598-019-46447-7

**Published:** 2019-07-10

**Authors:** Solbi Kim, Dongjoon Ko, Yunhee Lee, Seonghui Jang, Younghoon Lee, Ill Young Lee, Semi Kim

**Affiliations:** 1 Immunotherapy Research Center, Korea Research Institute of Bioscience and Biotechnology, Daejon, 34141 Korea; 2 Department of Functional Genomics, Korea University of Science and Technology, Daejon, 34113 Korea; 3 Department of Chemistry, Korea Advanced Institute of Science and Technology, Daejon, 34141 Korea; 4 Division of Drug Discovery Research, Korea Research Institute of Chemical Technology, Daejon, 34114 Korea

**Keywords:** Cancer, Target validation

## Abstract

Elevated expression of transmembrane serine protease 4 (TMPRSS4) correlates with poor prognosis in non-small cell lung cancer, gastric cancer, colorectal cancer, prostate cancer, and other cancer patients. Previously, we demonstrated that TMPRSS4 mediates tumor cell invasion, migration, proliferation, and metastasis. In addition, we reported novel 2-hydroxydiarylamide derivatives, IMD-0354 and KRT1853, as TMPRSS4 serine protease inhibitors. Here, we further evaluated the effects of the representative derivatives on TMPRSS4-mediated cellular function and signaling. IMD-0354 and KRT1853 inhibited cancer cell invasion, migration, and proliferation in TMPRSS4-expressing prostate, colon, and lung cancer cells. Both compounds suppressed TMPRSS4-mediated induction of Sp1/3, AP-1, and NF-κB transcription factors. Furthermore, TMPRSS4 promoted cancer cell survival and drug resistance, and both compounds enhanced anoikis sensitivity as well as reduced bcl-2 and survivin levels. Importantly, KRT1853 efficiently reduced tumor growth in prostate and colon cancer xenograft models. These results strongly recommend KRT1853 for further development as a novel anti-cancer agent.

## Introduction

Abnormal regulation of proteases is a key trait of tumor progression. Extracellular proteolytic enzymes, including matrix metalloproteinases and serine proteases, are involved in the development and progression of cancer, particularly in cancer cell invasion and metastasis, via not only direct proteolytic activity but also the regulation of cellular signaling and function^1,2^. Over ten years ago, Type II transmembrane proteases (TTSPs) were recognized as a new subfamily of serine proteases. All 17 members of the TTSP subfamily have, in common, a short cytoplasmic N-terminal domain, a transmembrane domain, and an extracellular proteolytic domain^3–5^. Most TTSPs are overexpressed in various tumors compared to normal corresponding tissues, indicating their potential as novel markers in tumor development and progression^4^. So far, many studies have analyzed the expression of individual TTSPs during tumor progression and focused on the investigation of the potential roles of these proteases in tumor cell migration, invasion, and proliferation^4,6^. For example, matriptase/MT-SP1 is overexpressed in a wide range of epithelial tumors including breast, ovary, uterus, prostate, cervix, and skin^6^. Matriptase is required for invasion *in vitro*, and causes spontaneous squamous cell carcinoma in a skin transgenic mouse model^7^, primarily through the hepatocyte growth factor (HGF) and proteinase-activated receptor (PAR)-2 signaling pathways^8^. Further, hepsin, another TTSP, is expressed in prostate, ovarian, and breast cancers and is required for tumor growth; however, the underlying cellular signaling is still under investigation^6^.

TMPRSS4, which was initially referred to as TMPRSS3^9^, is strongly upregulated in a variety of cancers. Elevated TMPRSS4 expression is associated with poor prognosis in non-small cell lung cancer with squamous cell histology, triple-negative breast cancer, cervical cancer, gastric cancer, colon cancer, and prostate cancer patients^10,11^. Previously, we reported that TMPRSS4 plays important roles in tumor cell migration, invasion, and metastasis, and that enhanced expression of TMPRSS4 is associated with colorectal cancer stage progression^12–14^. We also showed that TMPRSS4 upregulates the expression of the urokinase-type plasminogen activator (uPA) gene through activator protein-1 (AP-1) and Sp1/3 transcription factors and induces processing of pro-uPA into the active form to promote cancer cell invasion^15,16^. Furthermore, we demonstrated that TMPRSS4 induces both the invasion and proliferation of cancer cells through AP-1 activation and subsequent upregulation of Slug and cyclin D1^11^. These reports suggest that TMPRSS4 may serve as a potential molecular target for anti-cancer therapy and that inhibition of TMPRSS4 may have potential as a therapeutic strategy to reduce tumor growth as well as invasion and metastasis. Previously, we screened a small molecule compound library and identified novel 2-hydroxydiarylamide derivatives that inhibit TMPRSS4 serine protease activity and suppress invasion of colon cancer cells^14^. Among the derivatives, IMD-0354 (N-(3,5-Bis-trifluoromethylphenyl)-5-chloro-2-hydroxybenzamide) is a selective IκB kinase (IKK) β inhibitor that blocks phosphorylation of IκBα, thus preventing nuclear translocation and activation of NF-κB. Additionally, IMD-0354 is effective in acute and subacute inflammatory disease^17^. Although IMD-0354 displayed relatively potent inhibitory activity against TMPRSS4, KRT1853 (a novel derivative of IMD-0354 with bromine at the R′^2^ position) displayed two-fold higher inhibition^14^.

In this study, we explored the therapeutic potential of IMD-0354 and KRT1853 against TMPRSS4-expressing prostate, colon, and lung cancer cells and evaluated the underlying mechanism of anti-tumor activity. IMD-0354 and KRT1853 efficiently reduced cancer cell invasion and proliferation as well as induced apoptosis. Importantly, KRT1853 efficiently reduced tumor growth *in vivo*. These results support TMPRSS4 as a potential molecular target for anti-cancer therapy and recommend KRT1853 for further development as a novel anti-cancer agent.

## Results

### TMPRSS4 promotes cancer cell survival through upregulation of bcl-2 and survivin

Previously, we reported that TMPRSS4 promotes prostate cancer cell invasion and proliferation through activation of Sp1 and AP-1 transcription factors and subsequent upregulation of Slug and cyclin D1^11,15^. These previous findings led us to anticipate that TMPRSS4 may modulate cancer cell survival.

**Figure 1:**
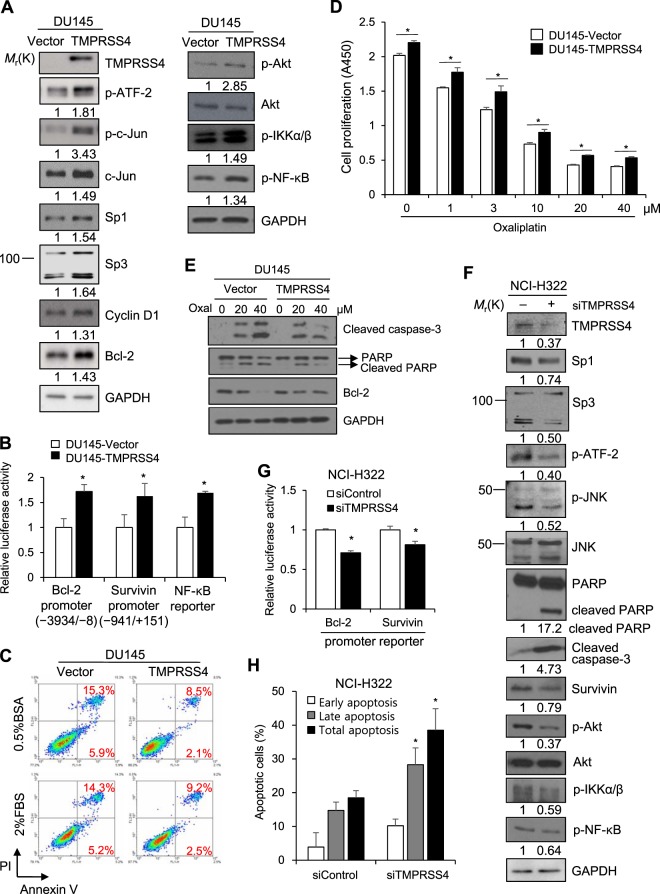
TMPRSS4 upregulates bcl-2 and survivin and promotes cancer cell survival. (**A**) DU145 cells were stably transfected with a TMPRSS4 expression vector or empty vector. Cells were lysed and used for immunoblotting. An anti-myc antibody was used to detect myc-tagged TMPRSS4. GAPDH was used as an internal control. (**B**) DU145 stable cells were transfected with NF-κB reporter or bcl-2 (−3934/−8) or survivin (−941/+151) promoter reporter constructs. Firefly luciferase activity, representing NF-κB transcriptional activity, bcl-2 promoter activity, or survivin promoter activity, was measured after 48 h and normalized to Renilla luciferase activity to determine the transfection efficiency. (**C**) DU145 stable cells were incubated for 48 h with 2% FBS or 0.5% bovine serum albumin (BSA) under suspension culture conditions and then stained with annexin V and PI for flow cytometric analysis. (**D**) DU145 stable cells were treated with oxaliplatin for 48 h. Cell viability was determined by the colorimetric WST assay. DMSO (0.1%) was used as a vehicle. (**E**) DU145 stable cells were treated with oxaliplatin for 48 h prior to lysis for immunoblotting. (**F**) NCI-H322 cells were transiently transfected with TMPRSS4-specific siRNA for 48 h prior to lysis for immunoblotting. Densitometry quantification was performed on the immunoblots using GAPDH as a loading control except that phospho-Akt and phospho-JNK were normalized against the corresponding total protein levels. (**G**) NCI-H322 cells were co-transfected with siRNA and promoter reporter constructs for 48 h. Luciferase activity was measured as in (**B**). (**H**) NCI-H322 cells were transfected with siRNA for 48 h and then stained with annexin V and PI. Stained cells relative to total cells were counted to calculate the percentage of apoptotic cells. Values represent mean ± standard deviation (SD). *P < 0.05.

To examine TMPRSS4-mediated cellular function and activity of TMPRSS4 inhibitors, we first generated stable TMPRSS4-overexpressing DU145 prostate cancer cell lines. As expected, TMPRSS4-overexpressing DU145 cells displayed elevated invasiveness and migratory activity compared with vector-transfectants (Fig. S1A,B). TMPRSS4 overexpression significantly enhanced proliferation of DU145 cells by 24% over 48 h (Fig. S1C). Moreover, the reporter activities of AP-1, CRE, and Sp1 were elevated in TMPRSS4-overexpressing DU145 cells compared to vector-transfectants (Fig. S1D). Using HCT116 and SW620 cells, we again demonstrated that TMPRSS4 promoted cancer cell migration and invasion while TMPRSS4 moderately modulated cancer cell proliferation in a cell type- or context-dependent manner (Fig. S1E,F), which is consistent with our previous results^11,12,15^. Immunoblot analysis revealed that phosphorylation of c-Jun and ATF-2, as well as expression of Sp1, Sp3, and cyclin D1, were elevated in TMPRSS4-overexpressing DU145 stable cells compared with vector-transfectants (Fig. 1A). This finding was consistent with our previous observations in DU145 and PC3 prostate cancer cells that were transiently transfected with TMPRSS4^15^. In addition, TMPRSS4 upregulated bcl-2 expression and the phosphorylation of Akt, IKKα/β, and NF-κB (Fig. 1A). Reporter assays demonstrated that TMPRSS4 induced a 1.72-fold and 1.63-fold increase in bcl-2 (−3934/−8) and survivin (−941/+151) promoter activity (Fig. 1B) at 48 h post-transfection, respectively; however, survivin was not detected by immunoblot analysis. TMPRSS4 also induced a 1.69-fold increase in NF-κB transcriptional activity (Fig. 1B).

We then examined the survival of TMPRSS4-overexpressing cells under suspension culture conditions. Cells were incubated in the presence or absence of serum in low-attachment plates for 48 h, and the induction of anoikis sensitivity was measured by flow cytometry analysis to determine the percentage of apoptotic cells. TMPRSS4-overexpresing cells displayed lower apoptosis level than vector-transfectants (Fig. 1C). In addition, when cells were treated with oxaliplatin for 48 h, TMPRSS4-overexpressing cells displayed lower drug sensitivity compared to vector-transfectants (IC_50_ = 6.0 μM vs. 7.6 μM) (Fig. 1D). Immunoblot analysis showed that oxaliplatin-mediated cleavage of caspase-3 and PARP as well as reduction of bcl-2 were attenuated in TMPRSS4-overexpressing cells compared with vector-transfectants (Fig. 1E).

We also examined the effect of endogenous TMPRSS4 on cell survival. Knockdown of TMPRSS4 in NCI-H322 cells, which display intermediate endogenous TMPRSS4 expression (Fig. S1A), by siRNA downregulated expression of Sp1 and Sp3 as well as phosphorylation of JNK and ATF-2, as observed previously^15^. Further, suppression of TMPRSS4 reduced expression of survivin and phosphorylation of Akt, IKKα/β, and NF-κB as well as enhanced the cleavage of PARP and caspase-3 (Fig. 1F). TMPRSS4 knockdown also significantly decreased bcl-2 and survivin promoter activities (Fig. 1G). Additionally, TMPRSS4 knockdown increased the percentage of apoptotic cells (Fig. 1H). Together, these results suggest that TMPRSS4 promotes cancer cell survival and drug resistance, potentially through upregulation of bcl-2 and survivin.

### IMD-0354 and KRT1853 inhibit cancer cell invasion and proliferation

**Figure 2:**
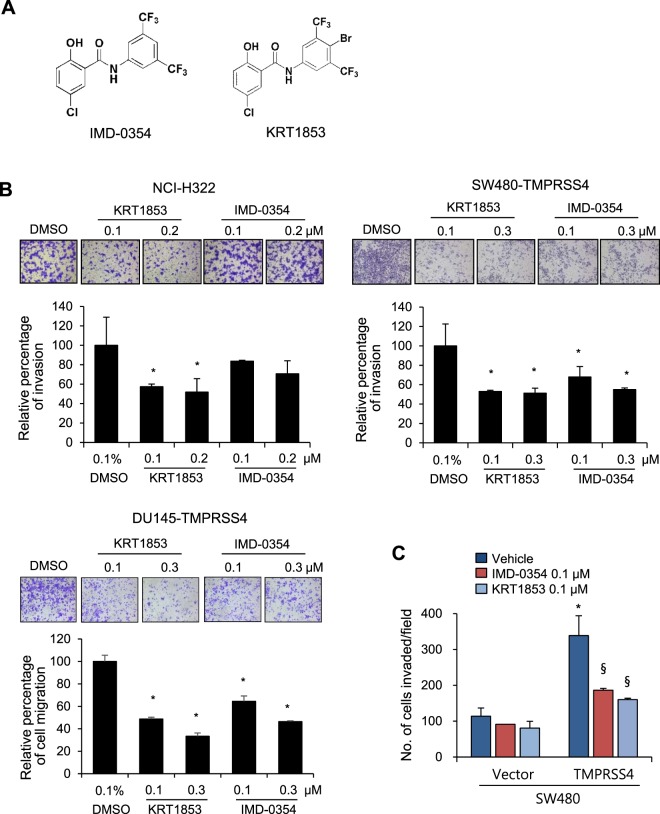
IMD-0354 and KRT1853 inhibit cancer cell invasion. (**A**) Structure of IMD-0354 and KRT1853. (**B**) NCI-H322 cells (2.5 × 10^4^) and TMPRSS4-overexpressing SW480 (4 × 10^4^) and DU145 cells (6 × 10^3^) were allowed to invade Matrigel or migrate for 48 h in the presence of compounds. The number of cells that invaded or migrated was counted in five representative (×100) fields per Transwell insert. Values represent mean ± SD. *P < 0.05. (**C**) TMPRSS4-overexpressing SW480 cells and vector transfectants (4 × 10^4^) were subjected to invasion assay in the presence of the compound at a concentration of 0.1 μM for 48 h. Values represent mean ± SD. *P < 0.05 compared with vector + vehicle; ^§^*P* < 0.05 compared with TMPRSS4 + vehicle.

**Figure 3:**
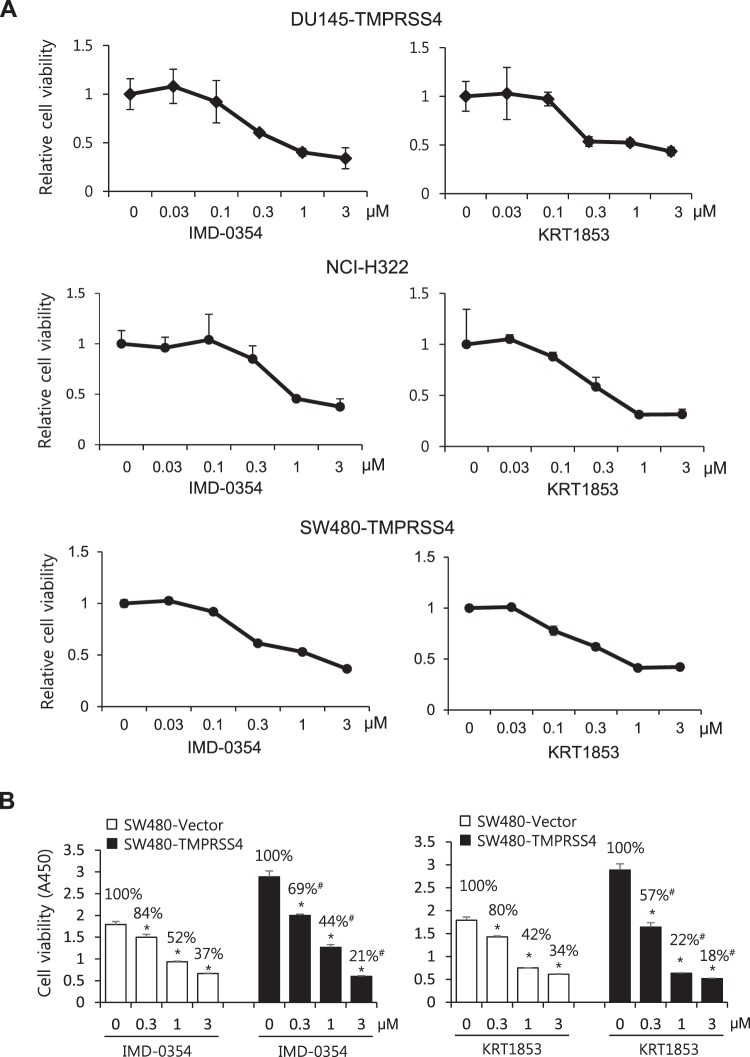
IMD-0354 and KRT1853 inhibit cancer cell proliferation. (**A**) Cells were seeded onto 96-well plates at a density of 5,000 cells/well and then incubated with the compounds for 48 h. Cell viability was determined by the colorimetric WST assay. (**B**) SW480 stable cells were seeded in the presence of the compounds for 48 h. Relative cell viabilities (%) per stable cell line are shown in the graph above. Values represent mean ± SD. *P < 0.05 compared with vehicle; ^#^*P* < 0.05 compared with the viability percentage of vector-transfected cells treated with corresponding concentrations of the compounds.

Previously, we reported that novel 2-hydroxydiarylamide derivatives inhibited TMPRSS4 serine protease activity and efficiently suppressed invasion of colon cancer cells overexpressing TMPRSS4^14^. Here, we evaluated the inhibitory activity and the underlying mechanism of two representative derivatives, IMD-0354 and KRT1853 (Fig. 2A), on invasion and proliferation of prostate, colon, and lung cancer cells. IMD-0354 and KRT1853 inhibited migration or invasion of TMPRSS4-overexpressing DU145 and SW480 cells and endogenous TMPRSS4-expressing NCI-H322 cells at sub-micromolar concentrations. In general, KRT1853 displayed more efficient inhibitory activity than IMD-0354 (Fig. 2B). Of note, KRT1853 or IMD-0354 efficiently inhibited invasion of TMPRSS4-overexpressing SW480 cells, whereas either compound did not significantly inhibit invasion of vector-transfected SW480 cells (Fig. 2C). As parental or vector-transfected SW480 cells normally display little endogenous TMPRSS4 expression, the inhibitory activity of the compounds likely depends on the level of TMPRSS4. Both compounds substantially suppressed the proliferation of TMPRSS4-expressing cells when cells were treated for 48 h at a higher concentration than that effective on invasion (Fig. 3A). In addition, compared with vector-transfected cells, TMPRSS4-overexpressing cells were relatively more sensitive/responsive to KRT1853 and IMD-0354 (Figs 3B and S2A), suggesting that the cytotoxicity of the compounds is, at least in part, dependent on TMPRSS4 expression. Consistently, the reduced migration of TMPRSS4-overexpressing DU145 stable cells upon KRT1853 or IMD-0354 treatment was increased by heightened TMPRSS4 overexpression by transient transfection (Fig. S2B).

### IMD-0354 and KRT1853 induce cancer cell apoptosis

**Figure 4:**
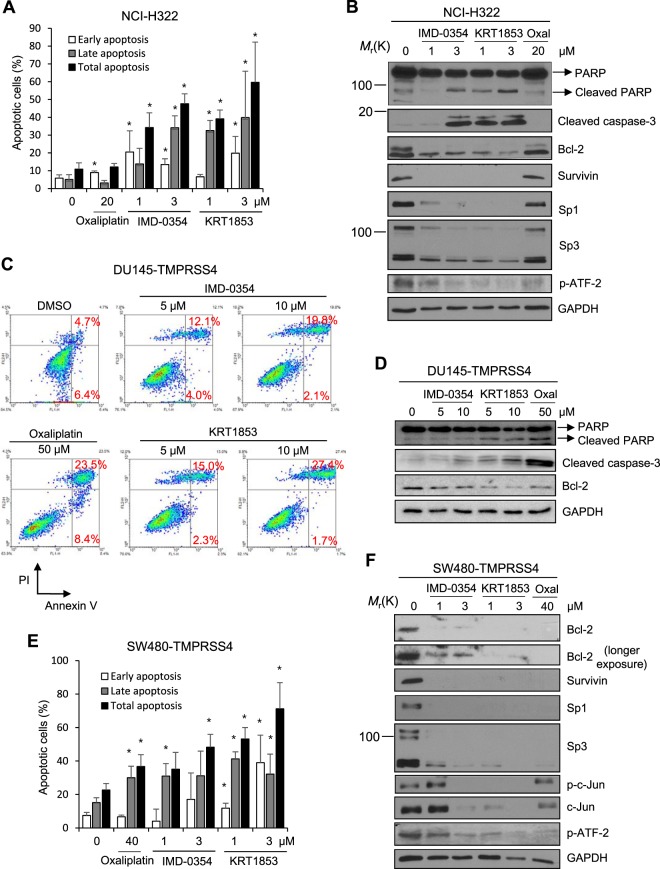
IMD-0354 and KRT1853 promote cancer cell apoptosis. (**A**,**B**) NCI-H322 cells were incubated with compounds for 48 h. (**A**) Cells were then stained with annexin V and PI. Stained cells relative to total cells were counted to calculate the percentage of apoptotic cells. (**B**) Cells were then lysed for immunoblotting. (**C**,**D**) TMPRSS4-overexpressing DU145 cells were treated with compounds for 48 h under suspension conditions. (**C**) Cells were then stained with annexin V and PI for flow cytometric analysis. (**D**) Cells were then lysed for immunoblotting. (**E**,**F**) TMPRSS4-overexpressing SW480 cells were treated with compounds for 48 h. (**E**) Cells were then stained with annexin V and PI. Stained cells relative to total cells were counted to calculate the percentage of apoptotic cells. Values represent mean ± SD. *P < 0.05. (**F**) Cells were then lysed for immunoblotting.

We also examined whether KRT1853 and IMD-0354 influenced the survival of TMPRSS4-positive cancer cells. When NCI-H322 cells were treated with the compounds for 48 h, apoptosis was increased by KRT1853 and IMD-0354 at 1~3 μM. Interestingly, KRT1853 and IMD-0354 induced a more prominent apoptotic response compared with a higher concentration of oxaliplatin (Fig. 4A). Immunoblot analysis showed that KRT1853 and IMD-0354 induced cleavage of PARP and caspase-3 more efficiently. Further, bcl-2, survivin, Sp1/3, and phospho-ATF-2 were all downregulated by KRT1853 and IMD-0354 (Fig. 4B). The effect of the compounds on reduction of anti-apoptotic factors was also more efficient than oxaliplatin. Treatment of TMPRSS4-overexpressing DU145 cells in suspension with KRT1853 and IMD-0354 at 5~10 μM resulted in increased anoikis in a dose-dependent manner (Fig. 4C), which was accompanied by an induction of cleaved caspase-3 and PARP as well as reduction of bcl-2 (Fig. 4D). When TMPRSS4-overexpressing SW480 cells were incubated with the compounds, apoptosis was enhanced at 1~3 μM (Fig. 4E). In addition, KRT1853 treatment drastically reduced bcl-2, whereas IMD-0354 reduced bcl-2 to a lesser extent in TMPRSS4-overexpressing SW480 cells (Fig. 4F). Survivin, Sp1/3, and phosphorylation of ATF-2 and c-Jun were also downregulated (Fig. 4F).

### IMD-0354 and KRT1853 suppress TMPRSS4-mediated signaling activity

**Figure 5:**
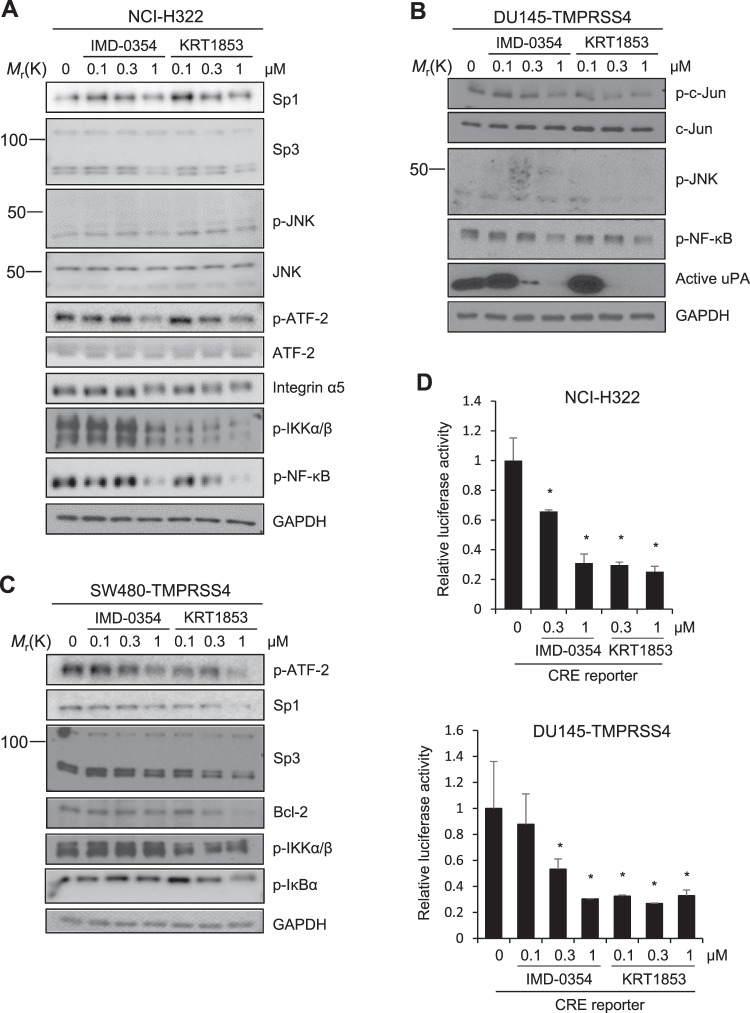
IMD-0354 and KRT1853 partially suppress TMPRSS4-mediated signaling activity. (**A**) NCI-H322 cells were treated with compounds for 24 h prior to lysis for immunoblotting. (**B**) TMPRSS4-overexpressing DU145 cells were treated with compounds for 24 h before whole-cell lysates were prepared. Conditioned medium was collected for an additional 48 h for immunoblotting. (**C**) TMPRSS4-overexpressing SW480 cells were treated with compounds for 24 h prior to lysis. (**D**) Cells were transfected with CRE reporter plasmid for 24 h. Transfected cells were treated with compound or vehicle for 24 h before lysis. Luciferase activity was measured as in Fig. 1B. Values represent mean ± SD. *P < 0.05.

We then explored whether IMD-0354 and KRT1853 suppressed TMPRSS4-mediated signaling activity. Cells were treated with the derivatives at concentrations of up to 1 μM for 24 h, which did not induce substantial apoptosis. In NCI-H322 cells, immunoblot analysis showed that KRT1853 and IMD-0354 treatment reduced the expression of Sp1, Sp3, and integrin α5 as well as phosphorylation of JNK (moderately) and ATF-2. Phosphorylation of IKKα/β and NF-κB was also downregulated by KRT1853 (substantially) and IMD-0354 in a dose-dependent manner (Fig. 5A), which was expected because IMD-0354 is a known IKKβ inhibitor.

In TMPRSS4-overexpressing DU145 cells, KRT1853 reduced phosphorylation of JNK and c-Jun, whereas IMD-0354 moderately reduced phosphorylation of c-Jun. Phosphorylation of NF-κB was also downregulated by both derivatives. Immunoblot analysis of conditioned media showed that production of active uPA was also reduced by both derivatives (Fig. 5B). In TMPRSS4-overexpressing SW480 cells, phosphorylation of ATF-2 and expression of Sp1 and Sp3 were reduced by KRT1853 and IMD-0354, whereas bcl-2 was substantially reduced by KRT1853 (Fig. 5C). Notably, at higher concentrations, which substantially induce cell apoptosis, the inhibitory effects of the compounds on signaling activity were more prominent (Fig. 4B,F). Phosphorylation of IKKα/β and IκBα was reduced by KRT1853 (Fig. 5C). Consistently, KRT1853 and IMD-0354 significantly reduced CRE reporter activity in NCI-H322 and TMPRSS4-overexpressing DU145 cells (Fig. 5D).

We previously reported that IMD-0354 and KRT1853 exhibited IC_50_ values of 11 μM and 6 μM, respectively, against TMPRSS4 and were more efficient than the general serine protease inhibitor AEBSF, which exhibited an IC_50_ value of 39 μM^14^. Notably, the relatively high IC_50_ values of the compounds against TMPRSS4 serine protease activity *in vitro* compared with those against cancer cell invasion or viability may be partially due to the low specific activity of recombinant TMPRSS4 serine protease. Next, we evaluated the target selectivity of KRT1853 and IMD-0354 on other proteases. Both compounds failed to substantially inhibit caspase-3 (aspartic protease), cathepsin B (cysteine protease), DPP4, DPP9, FAP (serine proteases), or MMP2 (metalloprotease) protease activities with the exception of DPP4, which displayed an IC_50_ value of 9.2 μM IMD-0354 (Fig. S3). These results further confirm that KRT1853 and IMD-0354 selectively target TMPRSS4 at certain levels.

Together, these findings suggest that IMD-0354 and KRT1853 suppress TMPRSS4-mediated signaling activity and active uPA level, leading to inhibition of invasion and proliferation as well as the induction of apoptosis.

### KRT1853 efficiently inhibits tumor growth *in vivo*

**Figure 6:**
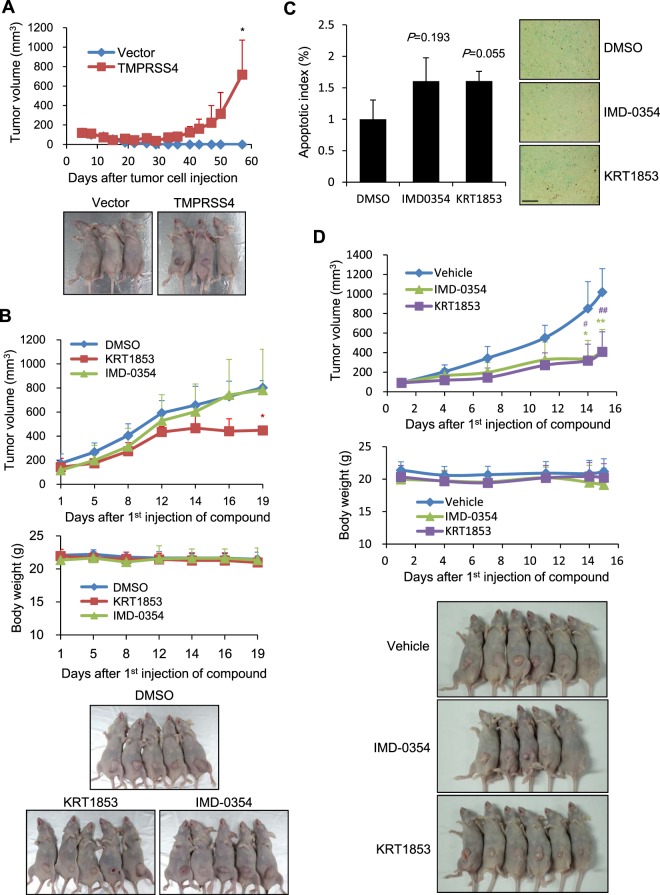
KRT1853 and IMD-0354 inhibit tumor growth *in vivo*. (**A**) TMPRSS4-overexpressing DU145 cells and vector transfectants (1 × 10^7^ cells/mouse) were subcutaneously injected into nude mice (n = 5) as described in the Materials and Methods. Tumor growth and body weight were measured for 8 weeks. Tumor volume was calculated using the formula, length × width^2^/2. Values of the maximum and minimum per group were excluded for the mean calculation. Values represent mean ± SD. *P = 0.025. (**B**) TMPRSS4-overexpressing DU145 cells were subcutaneously injected into nude mice (1 × 10^7^ cells/mouse). On day 40, tumor-bearing mice were randomized into control and treatment groups (n = 5 each). KRT1853 or IMD-0354 were injected into the tumors at intervals of 3 or 4 days for a total of six times. Upper: Tumor volume. Value of the maximum per group was excluded for the mean calculation. Values represent mean ± SD. *P = 0.0012. Middle: Body weight of injected mice. Lower: Photos of tumor-bearing mice on day 19 after compound injection. (**C**) TUNEL staining of tumor sections from (**B**) was performed to measure the level of apoptosis. Representative images are shown. The apoptosis index (%) was determined by calculating the number of TUNEL-positive cells relative to the total number of cells, which consisted of at least 1,000 cells per field. Five randomly selected fields of tumor sections per mouse were analyzed, excluding necrotic areas. Bar, 200 μm. Values represent mean ± SD. *P* value is shown above the graph. (**D**) HCT116 cells were subcutaneously injected into nude mice (5 × 10^6^ cells/mouse). On day 15, tumor-bearing mice were randomized into control and treatment groups (n = 5~6 per group). KRT1853 or IMD-0354 were intraperitoneally injected into the mice at intervals of 2 or 3 days for a total of six times. Upper: Tumor volume. Values represent mean ± SD. *P = 0.00631, **P = 0.00202, ^#^*P* = 0.00229, ^##^*P* = 0.00079. Middle: Body weight of injected mice. Lower: Photos of tumor-bearing mice on day 15 after compound injection.

When stable DU145 cells were injected into nude mice, tumor growth was significantly increased in mouse xenografts with TMPRSS4-overexpressing DU145 cells compared with vector-transfectants (Fig. 6A), consistent with *in vitro* results. We then examined the anti-tumor activity of KRT1853 and IMD-0354 in nude mice bearing TMPRSS4-overexpressing DU145 xenografts. Briefly, cells were injected subcutaneously into the flanks of nude mice. When tumor volumes reached approximately 150 mm^3^, vehicle or compound (15 μg/mouse/time) was intratumorally injected at 3- or 4-day intervals for a total of six times. KRT1853 significantly inhibited tumor growth by 47%, whereas IMD-0354 did not (Fig. 6B). Body weight was not affected by either compound (Fig. 6B). TUNEL staining of tumor sections showed that the level of apoptosis in tumors from mice injected with KRT1853 was higher than that in tumors from mice injected with vehicle although the difference showed marginal statistical significance at the level of *P* = 0.055 (Fig. 6C). Further, the level of apoptosis was not significantly different between tumors injected with IMD-0354 and vehicle. These results suggest that KRT1853 efficiently inhibits tumor growth in a prostate cancer xenograft model, potentially through the induction of apoptosis.

We also investigated the anti-tumor activity of KRT1853 and IMD-0354 in nude mice bearing endogenous TMPRSS4-expressing HCT116 colon cancer xenografts. As shown in Fig. 6D, administration of KRT1853 and IMD-0354 (400 μg/mouse/time) by intraperitoneal injection at 2- or 3-day intervals for a total of six times significantly inhibited tumor growth by 60% without affecting body weight. This result suggests that KRT1853 and IMD-0354 efficiently inhibit tumor growth in a colon cancer xenograft model. The anti-tumor efficacy of KRT1853 and IMD-0354 may depend on the cancer type and dose or pharmacokinetic properties of the compound. Further evaluation of the anti-tumor activity of these compounds against TMPRSS4-positive cancers *in vivo* is warranted.

## Discussion

TMPRSS4, a member of TTSP family, is highly expressed in pancreatic, thyroid, lung, colon, prostate, and other cancers^6,10^. We previously showed that TMPRSS4 enhances EMT and invasion of colon, prostate, and lung cancer cells^12,15^. We also reported that TMPRSS4 promotes proliferation of lung and prostate cancer cells via activation of AP-1 and Sp1^11,12^, indicating that TMPRSS4 is positively involved in both proliferation and invasion, although these are not common roles of TTSP family members. Similarly, a recent paper showed that TMPRSS4 promotes thyroid cancer cell proliferation via CREB phosphorylation^18^. Our previous observation that AP-1 and Sp1 are activated by TMPRSS4 led us to anticipate that TMPRSS4 may modulate cancer cell survival and that inhibition of TMPRSS4 may be an efficient therapeutic strategy for cancer treatment. In this study, we report that TMPRSS4 upregulates bcl-2 and survivin to enhance cancer cell survival, and inhibits anoikis and drug treatment sensitivity, potentially via upregulation of AP-1, Sp1, and NF-κB. IMD-0354 and its derivative KRT1853 displayed reduced TMPRSS4-mediated signaling activity, leading to suppression of invasion, proliferation, and survival of cancer cells. KRT1853 (prostate and colon cancer xenografts) and IMD-0354 (colon cancer xenograft) suppressed tumor growth *in vivo*, suggesting that a certain level of apoptosis induction and/or proliferation inhibition is required for anti-tumor effects *in vivo*. *In vitro*, KRT1853 induced cancer cell apoptosis more efficiently than oxaliplatin, suggesting that KRT1853 may be a more specific and/or efficient anti-cancer agent compared to conventional chemotherapeutic drugs. These results support that KRT1853 is a potential agent for the treatment of TMPRSS4-expressing cancers. As KRT1853 and IMD-0354 inhibited cancer cell survival and invasion, the evaluation of the metastasis-suppressing activity of these compounds *in vivo* is warranted.

Of note, IMD-0354 is a known selective IKKβ inhibitor that is effective in acute and subacute inflammatory disease^17^, and IMD-0354 did not exhibit substantial inhibitory activity against 102 other kinases at up to 10 μM (publicly available data). Therefore, it is probable that IMD-0354 and KRT1853 directly inhibit not only NF-κB but also TMPRSS4-mediated signaling activity including AP-1, Sp1, and NF-κB (indirectly). At present, we cannot explain how selective IKKβ inhibitor(s), such as IMD-0354, inhibit TMPRSS4 serine protease and related signaling activity. It would be worth investigating the structure of the TMPRSS4 inhibitor-docking protease domain. In terms of drug repositioning, KRT1853 and IMD-0354 would be useful anti-cancer agents based on guaranteed safety.

In general, TTSPs are viable targets for the development of therapeutic agents. Small molecule inhibitors, antibodies, and modified cognate inhibitors have been shown to effectively block the activity of several TTSPs and inhibit some aspects of cancer pathogenesis in cell and animal models^6^. For example, IN-1, a small molecule compound containing a ketobenzothiazole serine trap, is highly selective for matriptase and was shown to efficiently prevent cell proliferation and invasion^19^. SRI31215, a small molecule that acts as a triplex inhibitor of hepatocyte growth factor activator (HGFA), matriptase, and hepsin has been reported to inhibit EMT and migration of cancer cells^20^.

Our previous and present studies showed that KRT1853 and IMD-0354 directly inhibit TMPRSS4 serine proteolytic activity and TMPRSS4-mediated signaling. We expect that the primary target of KRT1853 and IMD-0354 in cancer cells used in the study is TMPRSS4 for two reason. First, the invasion-inhibitory and cell viability-inhibitory activities of the compounds were largely dependent on the TMPRSS4 expression level (Figs 2C and 3B), and, second, the activity of several other proteases was not substantially inhibited by the compounds (Fig. S3). However, at present, we cannot completely rule out the possibility that KRT1853 and IMD-0354 modulate the functions of other targets to regulate diverse cellular functions, including cell survival, proliferation, and invasion.

At present, it is not clear how TMPRSS4 activates signaling pathways, including JNK, and it is not clear how IMD-0354 and KRT1853 inhibit TMPRSS4-mediated signaling activity. Previously, we observed that TMPRSS4-mediated JNK activation is uPA receptor (uPAR)-dependent, and TMPRSS4 interacts with uPAR at the cell surface^15^. Thus, pro-uPA activation may be involved in TMPRSS4-uPAR-mediated signaling activity. It is possible that inhibition of TMPRSS4 serine proteolytic activity may directly and indirectly contribute to suppression of TMPRSS4-induced signaling activity.

It is intriguing that TMPRSS4 modulates cancer cell survival, proliferation, and invasion potentially through activation of major transcription factors. Previously, TMPRSS4 was known to activate JNK and ERK1/2, leading to activation of AP-1 and Sp1/3^11,15^. In this study, we observed that TMPRSS4 also activated NF-κB. Consistently, a recent paper reported that TMPRSS4 promotes invasion of gastric cancer cells through activation of NF-κB signaling^21^, although how NF-κB was activated by TMPRSS4 was not demonstrated. In our studies, TMPRSS4-mediated activation of FAK^13^ or Axl^11^ and subsequent Akt activation may be involved in NF-κB activation. In addition, it is possible that TMPRSS4 may be involved in the regulation of the tumor microenvironment, such as immune regulation or angiogenesis, via NF-κB to contribute to the accelerated development of aggressive malignancy. However, the mechanism of NF-κB activation by TMPRSS4 needs to be further investigated. It is well known that constitutive activation of NF-κB is a hallmark for leukemia, lymphoma, and solid tumors, and, thus, NF-κB is an important molecular target for new therapy strategies. Moreover, Sp-regulated genes are associated with cell survival, proliferation, angiogenesis, and metabolism^22^. Thus, Sp1 inhibitors, such as metformin^23^ or terameprocol^24^, may have therapeutic potential for cancer treatment. Therefore, TMPRSS4 may be an attractive target for potential anti-cancer therapy.

In summary, we evaluated two novel 2-hydroxydiarylamide derivatives as potential therapeutics against TMPRSS4-positive cancer cells. KRT1853, more efficiently, and IMD-0354 inhibited cancer cell proliferation and invasion as well as induced apoptosis. Both compounds inhibited TMPRSS4-mediated cellular signaling, including activation of Sp1, AP-1, and NF-κB and induction of bcl-2 and survivin. Importantly, KRT1853 efficiently suppressed tumor growth in nude mice bearing prostate and colon cancer xenografts. Altogether, this study demonstrates that TMPRSS4 is indeed a potential molecular target for anti-cancer therapy and recommends the development of KRT1853 as a novel anti-cancer agent.

## Methods

### Cell lines

Stable cells (vector-transfected and TMPRSS4-overexpressing cells) established from the SW480 cell line (colon cancer) were described previously^13^. DU145, PC3 (prostate cancer), HCT15, HCT116, SW620 (colon cancer), and NCI-H322 (lung cancer) cell lines were purchased from the American Type Culture Collection (ATCC; Manassas, VA, USA) and were maintained in RPMI1640 containing 10% fetal bovine serum (FBS) at 37 °C and 5% CO_2_. HeLa (cervical cancer) cells from ATCC were maintained in Eagle’s Minimum Essential Medium containing 10% FBS. Human embryonic kidney 293E (HEK293E) cells were purchased from ATCC and maintained in Dulbecco’s modified eagle medium (DMEM) containing 10% FBS. SNU-638 (gastric cancer) and SNU-398 (liver cancer) cell lines were purchased from the Korean Cell Line Bank (Seoul, Korea) and were maintained in RPMI1640 containing 10% FBS. Cell were checked for mycoplasma and their identities were confirmed using STR-PCR analysis.

### Generation of stable cell lines

DU145 cells were stably transfected with pCMV-myc-TMPRSS4^12^ or pCMV-Tag3 with Lipofectamine 2000 (Invitrogen, Carlsbad, CA, USA), according to the manufacturer’s instructions. G418-resistant clones were selected using 600 µg/ml of G418. The expression of myc-tagged TMPRSS4 was analyzed by immunoblotting.

### Transfection with small interfering RNA (siRNA)

Cells were transfected with siRNA specific to TMPRSS4 (5′-UCCAGUACGACAAACAGCACGUCUG-3′) using Lipofectamine 2000 for 48 h. At 48 h after transfection, cells were lysed for immunoblot analysis or harvested for apoptosis analysis.

### Immunoblot analysis

Whole-cell lysates were prepared using RIPA buffer, as described previously^15^, and analyzed using the following primary antibodies: anti-Sp1, anti-Sp3, anti-uPA, and anti-GAPDH (Santa Cruz Biotechnology, Santa Cruz, CA, USA); anti-myc (Upstate Biotechnology, Lake Placid, NY, USA); anti-phospho-c-Jun(S63), anti-c-Jun, anti-phospho-ATF-2(T71), anti-ATF-2, anti-phospho-JNK(T183/Y185), anti-JNK, anti-phospho-Akt, anti-Akt, anti-survivin, anti-bcl-2, anti-phospho-IKKα/β(S176/180), anti-phospho-IκBα(S32), anti-phospho-NF-κB p65(S536), anti-cleaved caspase-3, and anti-PARP (Cell Signaling Tech., Danvers, MA, USA); anti-integrin α5 (BD Biosciences, San Jose, CA, USA); and anti-TMPRSS4 (in-house)^15^. Where indicated, cells were treated with 0.1~10 μM KRT1853^14^ or IMD-0354 (Sigma, St Louis, MO, USA), 20~50 μM oxaliplatin (Sigma), or 0.1% dimethyl sulfoxide (DMSO) for 24~48 h before lysate preparation.

### Cell proliferation assay

Cell proliferation/viability was determined by the colorimetric WST assay (Ez-Cytox; Dogenbio, Seoul, Korea). Briefly, cells were seeded into 96-well plates at a density of 5,000 cells/well and incubated for 24 h. Cells were further incubated for 48 or 72 h in the presence of compound or vehicle. Where indicated, cells were seeded in the presence of compound for 48 h. Thereafter, cells were incubated with WST reagent (one-tenth of the medium volume) and the amount of formazan dye formed was determined by measuring absorbance at 450 nm using a spectrophotometric microplate reader (BMG LABTECH GmbH, Ortenber, Germany).

### Anoikis assay

Cells (5 × 10^5^) were seeded in the absence or presence of compound into 6-well plates with an Ultra-Low Attachment Surface (Corning, NY, USA) for 48 h to induce anoikis, as described previously^25^. Cells were washed and stained with 5 μl of annexin V and 5 μl of propidium iodide (PI) per 1 × 10^5^ cells for 15 min at room temperature in the dark, and the percentage of apoptotic cells was analyzed using flow cytometry. The cells were harvested after the induction of anoikis, washed with PBS, and lysed for immunoblot analysis. Where indicated, annexin V or PI-positive cells relative to the total cells were counted to determine the percentage of apoptotic cells.

### Promoter reporter assay

For transfection, cells were seeded into 6-well plates at a density of 2 × 10^5^ cells/well and incubated for 24 h. Cells were then transfected with 2 µg of reporter plasmids using Lipofectamine 2000. At 48 h post-transfection, firefly luciferase activity was measured using a Dual-luciferase reporter assay system (Promega, Southampton, UK). The transfection efficiency was normalized by measuring Renilla luciferase activity, encoded by the co-transfected Renilla luciferase vector (pRL-TK). For siRNA transfection, cells were co-transfected with siRNA and plasmid for 48 h. The bcl-2 (−3934/+8 relative to ATG) and survivin (−941/+151) promoter constructs were kindly provided by Addgene (#15381) and Dr. Gerhard Erkel^26^, respectively. The Sp1 cis-element reporter plasmid in which the Sp1 consensus site (GC-rich motif) and the TATA box were cloned into the luciferase reporter plasmid pGL2 (TATA-Sp1 reporter), was a kind gift from Dr. Yann Leverrier (Inserm, France)^27^. The AP-1, cyclic AMP response element (CRE), and NF-κB cis-element reporter plasmids were purchased from Stratagene (La Jolla, CA, USA). Where indicated, transfected cells were treated with compound or vehicle for 24 h before lysis.

### Invasion and cell migration assays

Invasion and cell migration assays were performed as described previously^12^. For the invasion assays, cells were plated in the presence of compound in serum-free medium on Transwell inserts (Corning, NY, USA) coated with 25 µg of Matrigel. The underside of the insert was pre-coated with 2 µg of collagen type I (Sigma). After incubation for 48 h at 37 °C and 5% CO_2_, inserts were fixed with 10% formalin and stained with 2% crystal violet. The number of cells that had invaded was counted in five representative (**×**100) fields per insert. Cell migration assays were performed in a similar manner but without the Matrigel coating.

### Protease activity assays

The protease screening and profiling service was performed by BPS Bioscience (San Diego, CA, USA). Inhibitory activities of KRT1853 and IMD-0354 against caspase-3, cathepsin B, dipeptidyl peptidase 4 (DPP4), DPP9, fibroblast activation protein (FAP), and matrix metalloproteinase 2 (MMP2) proteases were determined using a concentration range of 0.001~30 µM in 1% DMSO. An IC_50_ value of a positive control (reference compound) for each protease was also determined in parallel.

### Mouse xenograft models

All animal procedures were performed in accordance with the guidelines of the Animal Care Committee at the Korea Research Institute of Bioscience and Biotechnology (KRIBB). Animal experiment protocols were approved by the Animal Care Committee at KRIBB prior to experiments. Nude mice (BALB/c-nude, 5-week-old females) were obtained from Nara Biotech (Seoul, Korea). First, 1 × 10^7^ DU145 cells (vector and TMPRSS4-overexpressing stable cells) were injected subcutaneously into the right flank of each mouse (n = 5 per group). Body weight and tumor volume were measured twice a week for 8 weeks. Next, TMPRSS4-overexpressing DU145 stable cells (1 × 10^7^ cells) were injected subcutaneously into the right flank of each mouse. After 40 days, when tumor volumes reached approximately 150 mm^3^, the tumor-bearing mice were randomized into control and treatment groups (n = 5 per group). KRT1853 or IMD-0354 (15 µg/mouse) in 100 µl PBS was injected into the tumor of each mouse at 3- or 4-day intervals for a total of six times. Ten percent DMSO in 100 µl PBS was injected as a negative control. Body weight and tumor volume were measured prior to the compound injection. The tumor-bearing mice were sacrificed and photographed 19 days after 1^st^ injection of the compound. HCT116 cells (5 × 10^6^ cells) were injected subcutaneously into the right flank of each mouse. After 15 days, when tumor volumes reached approximately 100 mm^3^, the tumor-bearing mice were randomized into control and treatment groups (n = 5~6 per group). KRT1853 or IMD-0354 (400 µg/mouse) dissolved in 100 µl DMSO/Cremophor EL/PBS (1:1:8) was injected intraperitoneally into the mouse at 2- or 3-day intervals for a total of six times. Vehicle control was 100 µl DMSO/Cremophor EL/PBS (1:1:8). The tumor-bearing mice were sacrificed and photographed 15 days after 1^st^ injection of the compound. The tumor volumes were calculated as follows: tumor volume = (a × b^2^) × 1/2, where a was the width at the widest point of the tumor and b was the maximal width perpendicular to a. Tumor masses were fixed in 10% formalin and embedded in paraffin.

### TUNEL assay

TUNEL staining was performed to measure apoptosis in paraffin-embedded tumor sections using the ApopTag Plus Peroxidase *In Situ* Apoptosis Kit (Merck Millipore, Billerica, MA, USA) according to the manufacturer’s instructions^28^. Cell nuclei were stained with hematoxylin. The apoptosis index (%) was determined by calculating the number of TUNEL-positive cells relative to the total number of cells, which consisted of at least 1,000 cells per field.

### Statistical analysis

Statistical analyses were performed using the Student’s *t*-test. *P* < 0.05 was considered statistically significant.

## Data Availability

The datasets generated and/or analyzed during the current study are available from the corresponding author on reasonable request.

## Supplementary information

Supplementary Figures and Figure legends
