# Supplementary material for: Anti-cancer activity of the novel 2-hydroxydiarylamide derivatives IMD-0354 and KRT1853 through suppression of cancer cell invasion, proliferation, and survival mediated by TMPRSS4
Source: Sci Rep. 2019 Jul 10;9:10003. doi: 10.1038/s41598-019-46447-7 (PMC6620293; doi:10.1038/s41598-019-46447-7)
Supplement: Supplementary file 1 — Supplementary Figures and Figure legends [file 41598_2019_46447_MOESM1_ESM.pdf]

Anti-cancer activity of the novel 2-hydroxydiarylamide derivatives IMD-0354 and KRT1853 through suppression of cancer cell invasion, proliferation, and survival mediated by TMPRSS4

Solbi Kim, Dongjoon Ko, Yunhee Lee, Seonghui Jang, Younghoon Lee, Ill Young Lee, Semi Kim

Supplementary Figure Legends

Supplementary Figure S1. (A) Cells including DU145 and SW480 stable cells were lysed for the analysis of TMPRSS4 expression. Equal amounts of whole cell lysate (20 μg) were loaded, with the exception of the whole cell lysate (10 μg), from HEK293E cells transiently transfected with a TMPRSS4-expressing vector in lane 1 (Upper). (B) DU145 stable cells were allowed to invade Matrigel (1×10^4 cells) or migrate (6×10^3 cells) for 48 h. The number of cells that invaded or migrated was counted in five representative (×100) fields per Transwell insert. (C) DU145 stable cells were seeded into 96-well plates at a density of 5,000 cells/well and incubated for 48 h. Cell proliferation was determined by the colorimetric WST assay. (D) DU145 stable cells were transfected with reporter constructs. Firefly luciferase activity was measured after 48 h and normalized to Renilla luciferase activity to determine the transfection efficiency. (E) HCT116 cells were transfected with TMPRSS4-specific siRNA using electroporation for 48 h prior to lysis for immunoblotting. In parallel, transfected cells (2×10^4 cells) were subjected to invasion and cell migration assays for 48 h. Cell proliferation assay was also performed. (F) SW620 cells were transfected with a TMPRSS4 expression vector

using Lipofectamine 2000 for 48 h prior to lysis for immunoblotting. Anti-myc and anti-TMPRSS4 (in-house) antibodies were used to detect myc-tagged TMPRSS4. In parallel, transfected cells (1.5×10^5 cells) were subjected to invasion and cell migration assays for 48 h. Cell proliferation assay was also performed. Values represent mean ± standard deviation (SD). * $P < 0.05$.

Supplementary Figure S2. (A) TMPRSS4-overexpressing and vector-transfected DU145 cells were incubated with the compounds for 72 h. Cell viability was determined by the colorimetric WST assay. Relative cell viabilities (%) per stable cell line are shown in the graph above. Values represent mean ± SD. * $P < 0.05$ compared with vehicle; # $P < 0.05$ compared with the viability percentage of vector-transfected cells treated with corresponding concentrations of the compounds. (B) TMPRSS4-overexpressing DU145 cells were transiently transfected with a TMPRSS4-expressing vector or an empty vector using Lipofectamine 2000 for 48 h. Transfected cells were lysed for analysis of TMPRSS4 expression. For comparison, whole-cell lysates from vector-transfected DU145 stable cells were also loaded (lane 1). Anti-myc and anti-TMPRSS4 (in-house) antibodies were used to detect myc-tagged TMPRSS4. In parallel, transfected cells were subjected to the cell migration assay. The number of cells that migrated was counted in five representative (×100) fields per Transwell insert. Values represent mean ± SD. * $P < 0.05$ compared with vehicle; § $P < 0.05$ compared with vector transfection + treatment with compound at the corresponding concentration.

Supplementary Figure S3. Inhibitory effects of KRT1853 and IMD-0354 on protease activities. The IC_{50} of the compounds against proteases are summarized. If the IC_{50} was higher

than the highest test point (30 μM), the percent inhibition of the compound at 30 μM is reported. The IC_{50} of the reference compound for each protease was determined in parallel.

Supplementary Figure S1. Kim et al.

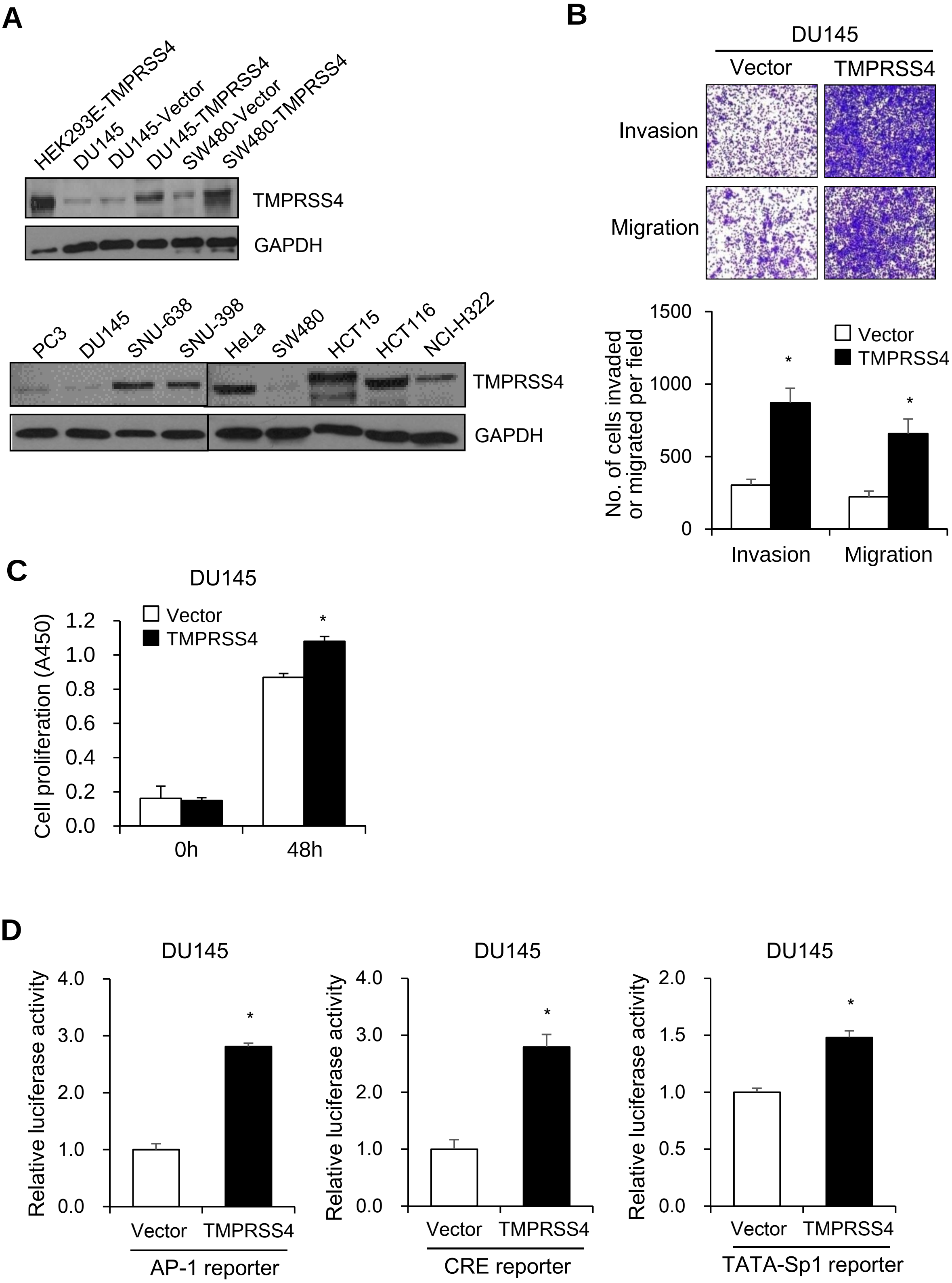

Supplementary Figure S1. Kim et al. (continued)

E

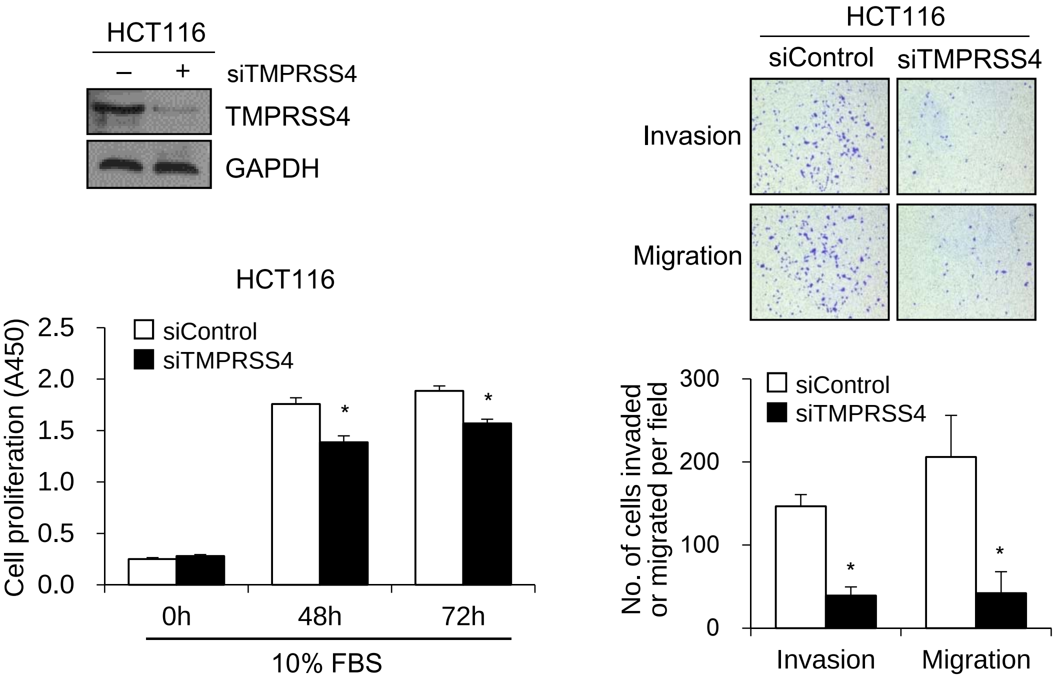

F

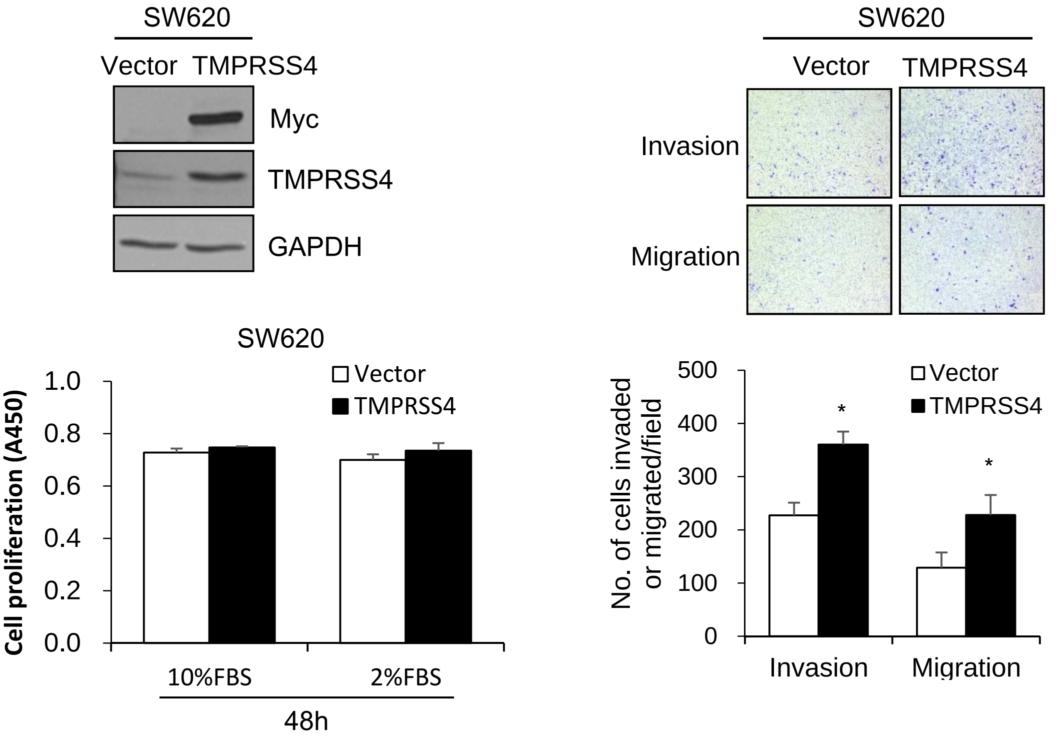

Supplementary Figure S2. Kim et al.

A

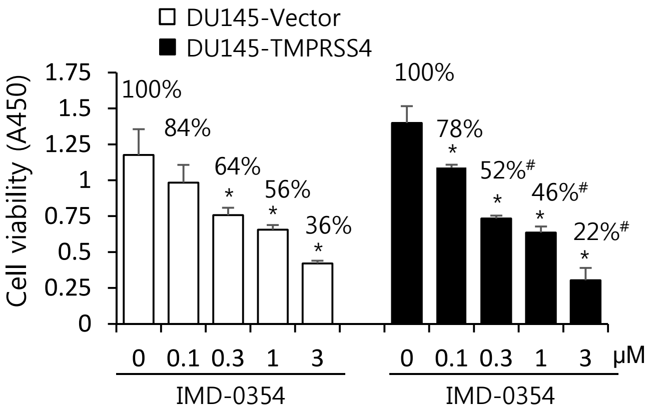

B

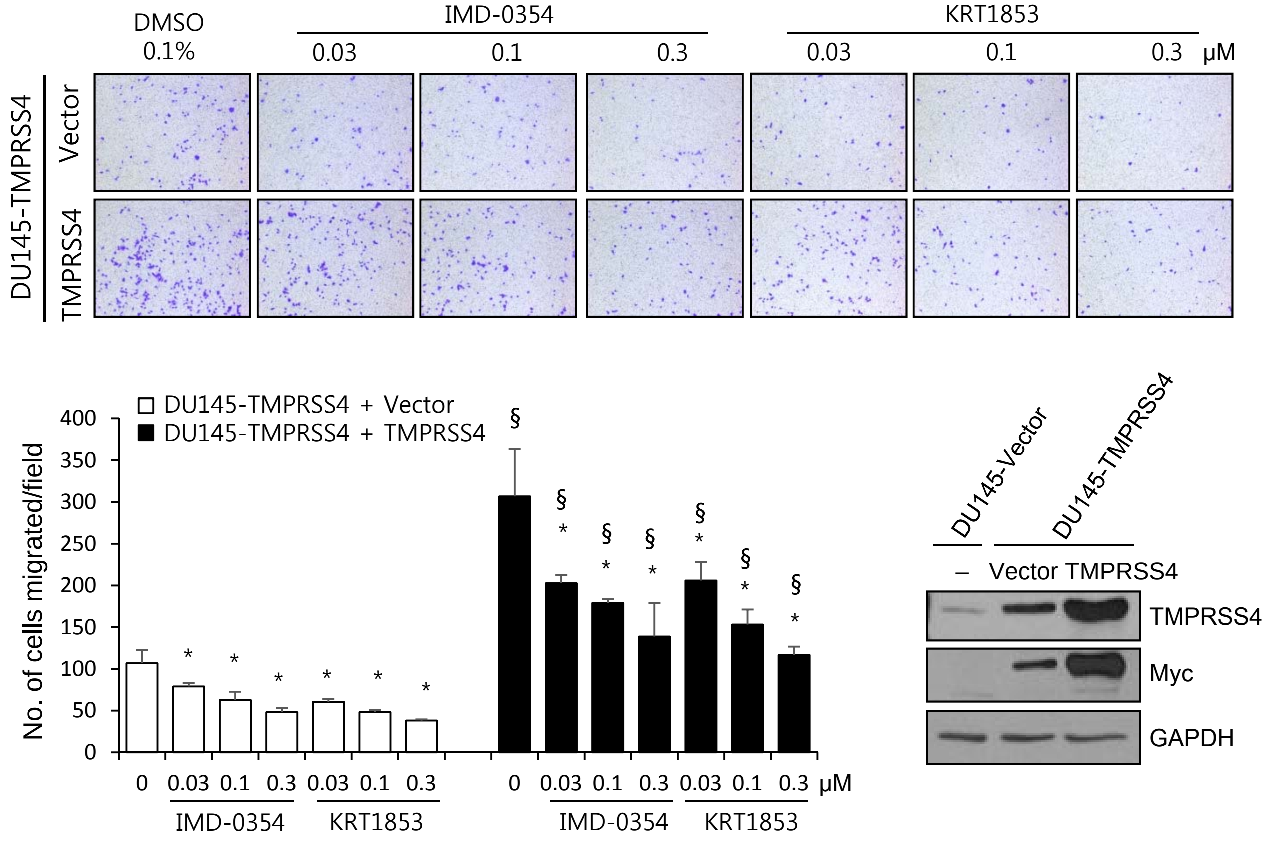

Supplementary Figure S3. Kim et al.

Enzyme	IC_{50} (μM) or Percentage inhibition (%)		
	KRT1853*	IMD-0354*	Reference
**Caspase-3**	IC_{50}>30μM<br>32% @ 30μM	IC_{50}>30μM<br>NI# @ 30μM	0.014<br>(Caspase 3/7 Inhibitor I)
**Cathepsin B**	IC_{50}>30μM<br>NI @ 30μM	IC_{50}>30μM<br>NI @ 30μM	0.002<br>(Cathepsin B Inhibitor II)
**DPP4**	IC_{50}>30μM<br>21% @ 30μM	9.2	0.014<br>(Sitagliptin)
**DPP9**	IC_{50}>30μM<br>25% @ 30μM	IC_{50}>30μM<br>28% @ 30μM	0.63<br>(KR62436)
**FAP**	IC_{50}>30μM<br>18% @ 30μM	IC_{50}>30μM<br>25% @ 30μM	0.0018<br>(SP-13786)
**MMP2**	IC_{50}>30μM<br>18% @ 30μM	IC_{50}>30μM<br>NI @ 30μM	0.004<br>(NNGH)

* Compounds have fluorescence background under some assay conditions.

NI denotes No significant Inhibition if the inhibition is less than fifteen percent.
